# The Use of the Perfusion Index to Predict Post-Induction Hypotension in Patients Undergoing General Anesthesia: A Systematic Review and Meta-Analysis

**DOI:** 10.3390/diagnostics14161769

**Published:** 2024-08-14

**Authors:** Kuo-Chuan Hung, Shu-Wei Liao, Chia-Li Kao, Yen-Ta Huang, Jheng-Yan Wu, Yao-Tsung Lin, Chien-Ming Lin, Chien-Hung Lin, I-Wen Chen

**Affiliations:** 1 Department of Anesthesiology, Chi Mei Medical Center, Tainan 71004, Taiwan; ed102605@gmail.com (K.-C.H.); buzzer176@gmail.com (S.-W.L.);; 2 Center of General Education, Chia Nan University of Pharmacy and Science, Tainan 71710, Taiwan; 3 Department of Anesthesiology, E-Da Hospital, I-Shou University, Kaohsiung 82445, Taiwan; 4 Department of Surgery, National Cheng Kung University Hospital, College of Medicine, National Cheng Kung University, Tainan 70403, Taiwan; 5 Department of Nutrition, Chi Mei Medical Center, Tainan 71004, Taiwan; 6 Department of Anesthesiology, Chi Mei Medical Center, Liouying, Tainan 73657, Taiwan

**Keywords:** predictive efficacy, perfusion index, general anesthesia, hypotension, propofol

## Abstract

Post-induction hypotension (PIH) is a common and potentially serious complication of general anesthesia. This meta-analysis (Prospero registration number: CRD42024566321) aimed to evaluate the predictive efficacy of the perfusion index (PI) for PIH in patients undergoing general anesthesia. A comprehensive literature search was performed using multiple electronic databases (Google Scholar, EMBASE, Cochrane Library, and MEDLINE). Studies involving adult patients undergoing general anesthesia, with the PI measured before anesthesia induction and reporting PIH incidence, were included. The primary outcome was the diagnostic accuracy of the PI in predicting the probability of PIH. The secondary outcome was the pooled PIH incidence. Eight studies with 678 patients were included. The pooled incidence of PIH was 44.8% (95% confidence interval [CI]: 29.9%–60.8%). The combined sensitivity and specificity of the PI for predicting PIH were 0.84 (95% CI: 0.65–0.94) and 0.82 (95% CI: 0.70–0.90), respectively. The summary receiver operating characteristic (sROC) analysis revealed an area under curve of 0.89 (95% CI: 0.86–0.92). The Deek’s funnel plot asymmetry test indicated no significant publication bias. The PI demonstrates high predictive efficacy for PIH in patients undergoing general anesthesia, indicating that it can be a valuable tool for identifying those at risk of PIH.

## 1. Introduction

Intraoperative hypotension is often characterized by a systolic blood pressure below 80–90 mmHg or a mean arterial pressure less than 60–65 mmHg [1,2]. Alternatively, it may be defined as a relative decrease of 20–30% from baseline blood pressure [1,2]. Intraoperative hypotension is a common occurrence, affecting up to 41–93% of patients undergoing noncardiac surgery, depending on the definition used [2]. Prolonged or severe hypotension can lead to the inadequate perfusion of vital organs, potentially resulting in ischemia and organ dysfunction [3]. Several retrospective studies have reported an association between intraoperative hypotension and an increased risk of acute kidney injury, myocardial injury, and stroke [4,5,6,7,8]. Therefore, the prevention, prompt recognition, and management of intraoperative hypotension are crucial for optimizing patient outcomes and reducing perioperative morbidity and mortality.

Compared to intraoperative hypotension, post-induction hypotension (PIH) is characterized by a drop in arterial blood pressure occurring within the first 20 min following anesthesia induction, or between the induction of anesthesia and the start of the surgical incision [9]. PIH is also common during anesthesia, with an incidence ranging from 18.1% to 70% [10,11]. Although the impact of PIH on postoperative outcomes remains to be clarified, a recent study reported that PIH may induce an increase in endogenous plasma catecholamines [11], potentially leading to postoperative complications, such as myocardial ischemia [12,13,14]. Consistently, a previous study found that patients with PIH had a higher incidence of prolonged postoperative stay and/or death than those without this condition [15]. Furthermore, a significant proportion of patients (i.e., 42%) with PIH may also experience intraoperative hypotension [16], emphasizing the need to prevent the occurrence of PIH.

The perfusion index is a noninvasive measure derived from pulse oximetry that reflects the ratio of pulsatile (i.e., arterial compartment) to non-pulsatile blood flow in the peripheral tissue. Recent studies have suggested that the perfusion index may serve as a valuable predictor of hemodynamic instability during anesthesia induction [17,18,19]. As a readily available parameter in most operating rooms, the perfusion index offers potential advantages over more invasive or time-consuming predictive methods. Nevertheless, some studies have found limited efficacy of pre-induction perfusion index values in predicting PIH [20,21]. The variability in study designs, patient populations, and definitions of PIH has made it difficult to draw definitive conclusions about the clinical utility of the perfusion index as a predictive tool in a single study. If proven effective, it could serve as a valuable tool for identifying patients at risk of PIH, enabling anesthesiologists to implement targeted preventive strategies and optimize patient care. To date, no comprehensive systematic review and meta-analysis has been conducted to synthesize available evidence on this topic. Therefore, this meta-analysis aimed to evaluate the predictive efficacy of the perfusion index for PIH in patients undergoing general anesthesia. 

## 2. Materials and Methods

### 2.1. Study Protocol

This review adhered to the PRISMA-DTA guidelines and was previously registered with PROSPERO (Registration number: CRD42024566321).

### 2.2. Data Source and Literature Search

A comprehensive literature search was conducted using multiple electronic databases, including Google Scholar, EMBASE, Cochrane Library, and MEDLINE, encompassing all studies available up to July, 2024. The search terms included combinations of keywords such as (“General anesthesia” OR “General anaesthesia” OR “Total intravenous anesthesia”) AND (“PI” OR “Perfusion index”) AND (“Hypotension” OR “Reduced blood pressure” OR “Low blood pressure”). Controlled vocabulary terms were used to ensure a thorough search. The reference lists of the retrieved articles were manually reviewed to identify additional relevant studies. There were no limitations on country, publication year, or language of publication. The search strategy for MEDLINE is presented in Table 1. Similar search strategies were employed in the other databases, with appropriate modifications based on the database-specific controlled vocabulary terms and syntax. Two independent researchers performed the search and screened the titles and abstracts of the identified articles. Full-text versions of potentially relevant studies were obtained and evaluated based on predefined criteria. Any disagreements were resolved through discussion by the same researchers, and a third researcher was consulted if necessary.

### 2.3. Inclusion and Exclusion Criteria

The inclusion criteria were as follows: prospective observational studies or randomized controlled trials involving adult patients undergoing general anesthesia for elective surgery, with the perfusion index measured before the induction of anesthesia and reporting the incidence of PIH. Only peer-reviewed articles were considered. The exclusion criteria were as follows: case reports, case series, review articles, editorials, and conference abstracts; studies involving pediatric patients; those focusing on general anesthesia combined with regional anesthesia; studies that did not provide a clear definition of PIH or did not measure the perfusion index before induction; studies with incomplete or unclear data regarding perfusion index measurements or PIH incidence; and duplicate publications or studies with overlapping patient populations.

### 2.4. Data Extraction 

Two independent reviewers extracted data from the included studies using a standardized form, collecting the following information: study characteristics (author(s) and year of publication, country of origin, sample size), patient demographics (age, sex distribution, American Society of Anesthesiologists (ASA) physical status), anesthesia details (type of surgery, anesthetic agents used for induction), perfusion index measurement details (timing of measurement relative to anesthesia induction, cut-off values used if applicable), PIH details (definition of PIH used in the study, incidence of PIH, time frame for PIH assessment after induction), outcome measures (sensitivity and specificity of perfusion index for predicting PIH). In cases of missing or unclear data, attempts were made to email the corresponding authors of the original studies. Any disagreements in data extraction between the two reviewers were resolved through discussion or consultation with a third reviewer.

### 2.5. Outcomes and Definitions

The primary outcome was the diagnostic accuracy of the perfusion index in predicting the probability of PIH. The secondary outcome was the pooled incidence of PIH.

### 2.6. Quality Assessment

We assessed the risk of bias and applicability concerns for each included study using the Quality Assessment of Diagnostic Accuracy Studies-2 (QUADAS-2) tool [22]. Two independent reviewers evaluated four key domains: patient selection, index test (perfusion index measurement), reference standard (post-induction hypotension), and flow and timing. For each domain, we judged the risk of bias and applicability concerns as “Low”, “High”, or “Unclear” based on specific criteria relevant to our review question. Disagreements between the reviewers were resolved through discussion or by consulting a third reviewer. 

### 2.7. Statistical Analysis

Statistical analysis was conducted using the MIDAS module in Stata 15 (StataCorp LLC, College Station, TX, USA). We calculated pooled sensitivity and specificity for the perfusion index in predicting PIH. A summary receiver operating characteristic (sROC) curve was constructed, and the area under the curve (AUC) was determined to evaluate the overall diagnostic performance of the PI. Heterogeneity among the included studies was assessed using the I^2^ statistic, with significant heterogeneity defined as an I^2^ value of >75%. We also conducted Fagan’s nomogram analysis to translate the diagnostic accuracy of the perfusion index into clinical practice, combining pre-test probabilities (25%, 50%, and 75%) with likelihood ratios to estimate post-test probabilities. Deek’s funnel plot asymmetry test was employed to assess the potential for publication bias, with a *p*-value < 0.05 indicating significant asymmetry.

## 3. Results

### 3.1. Study Selection and Study Characteristics

**Figure 1:**
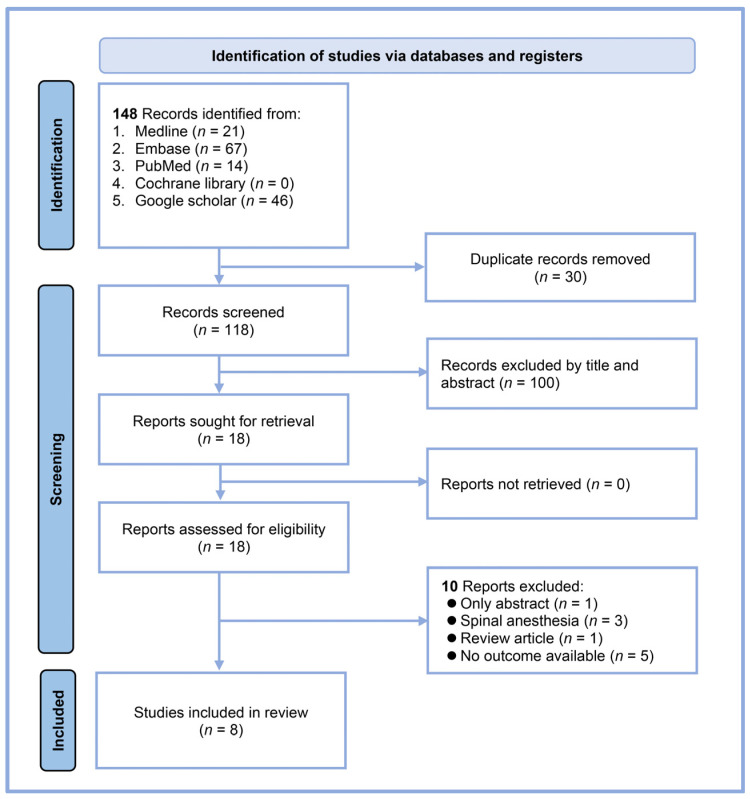
Flow diagram.

Our systematic search yielded 148 records from multiple databases: 21 from MEDLINE, 67 from Embase, 14 from PubMed, none from the Cochrane Library, and 46 from Google Scholar (Figure 1). After eliminating 30 duplicates, 118 unique articles were available for initial screening. We excluded 100 records based on the title and abstract review, leaving 18 reports for full-text assessment. A detailed evaluation of these full texts led to the exclusion of ten reports: one abstract-only study, three studies involving spinal anesthesia, one review article, and five studies that did not address our outcome of interest. Ultimately, eight studies met all the inclusion criteria and were included in our systematic review and meta-analysis [17,18,19,20,21,23,24,25]. All studies were conducted using a prospective design.

Our systematic review included eight studies with a total of 678 patients undergoing general anesthesia (Table 2). The studies were conducted in various countries: five from India, two from Egypt, and one from the Republic of Korea. The sample sizes ranged from 30 to 174 patients. The mean age of the participants varied across the studies, from 31 to 71 years. Most studies included patients with ASA physical status I-II, except for the study by Min et al. [25], which focused on patients with ASA II. All studies reported perfusion index cut-off values for predicting PIH, which ranged from 0.96 to 3.5. The AUC for predictive performance of the perfusion index varied from 0.511 to 1.0, indicating a wide range of predictive accuracies across studies. All studies used propofol for anesthesia induction (Table 3). In four studies, the doses ranged from 1 to 2 mg/kg [17,20,21,23]. Three studies titrated propofol at 10 mg every 5 s until loss of consciousness [18,19,24], while one study used a target effect-site concentration of 3 µg/mL [25]. The incidence of PIH varies widely among studies, ranging from 17.2% to 76.4% (Table 3). The definition of PIH varies across studies. Most studies used either a relative threshold (e.g., >30% drop from baseline) or an absolute threshold (e.g., MAP < 60 mmHg) for the systolic or mean arterial pressure (Table 3). Follow-up times for hypotension assessment ranged from 3 to 15 min post-induction, with 5 min being the most common. 

**Figure 2:**
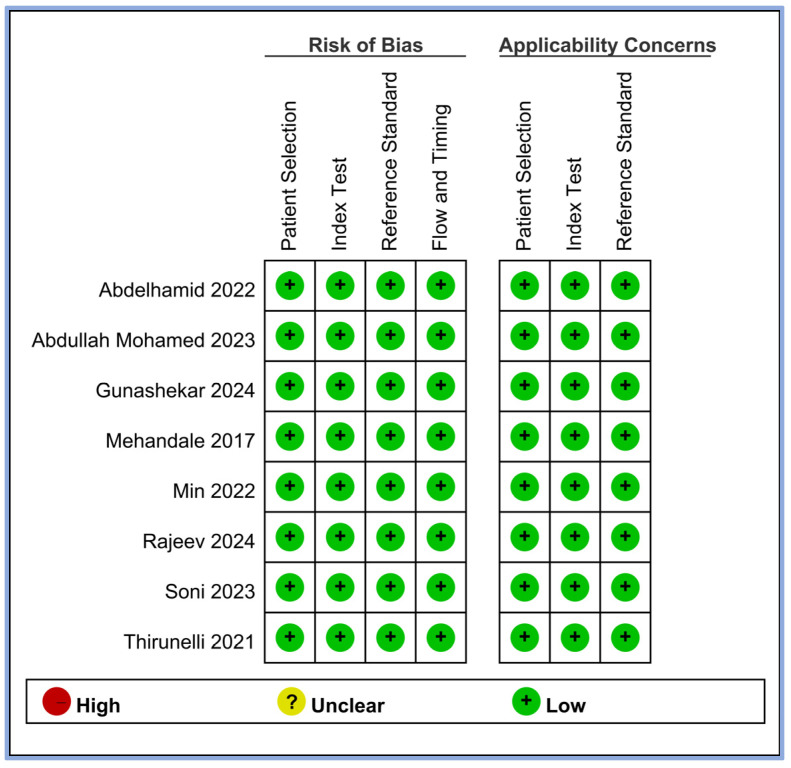
Methodological quality of the eight included studies [17,18,19,20,21,23,24,25].

We evaluated the risk of bias for all the included studies using four criteria: patient selection, index test, reference standard, and flow and timing. The results of this assessment are summarized in Figure 2. All eight studies demonstrated a low risk of bias across all four domains [17,18,19,20,21,23,24,25]. This consistently low risk suggests that the included studies were of high methodological quality in terms of how patients were selected, how the perfusion index (index test) was applied, how hypotension (reference standard) was defined and measured, and the timing and flow of patient assessments. In addition, we assessed the applicability concerns for patient selection, index tests, and reference standards. All studies showed low concerns regarding applicability in these three domains, indicating that the study population, perfusion index measurements, and definitions of hypotension were appropriate and relevant to our review question.

### 3.2. Outcomes

#### 3.2.1. Pooled Incidence of Post-Induction Hypotension

**Figure 3:**
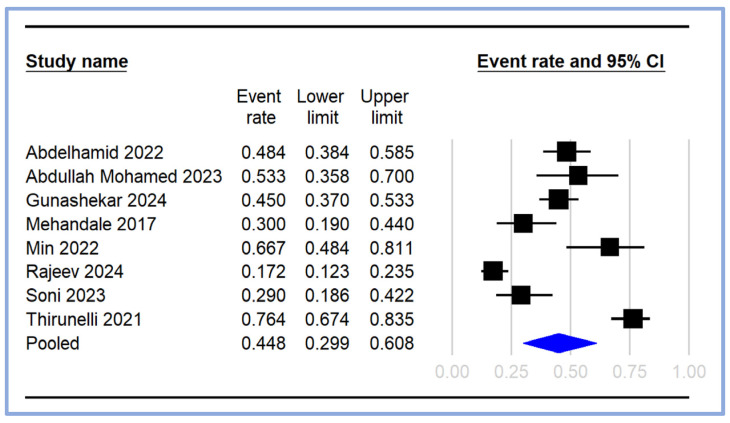
Pooled incidence of post-induction hypotension [17,18,19,20,21,23,24,25]. The event rates (squares) and their corresponding 95% CIs (horizontal lines) are presented for each study. The size of the squares reflects the weight of each study in the meta-analysis. The pooled event rate is represented by a diamond at the bottom of the plot, with the width of the diamond indicating the overall 95% CI. CI: confidence interval.

The pooled incidence of PIH was calculated from the included studies [17,18,19,20,21,23,24,25], with the event rates and 95% CI depicted in the forest plot (Figure 3). The individual study event rates varied significantly, ranging from 0.172 (95% CI: 0.123–0.235) [18] to 0.764 (95% CI: 0.674–0.835) [21]. The pooled event rate for PIH was estimated to be 0.448 (95% CI: 0.299–0.608), indicating that approximately 44.8% of the patients undergoing general anesthesia experienced hypotension following induction (Figure 3). 

#### 3.2.2. Pooled Sensitivity, Specificity, and Area under Curve

**Figure 4:**
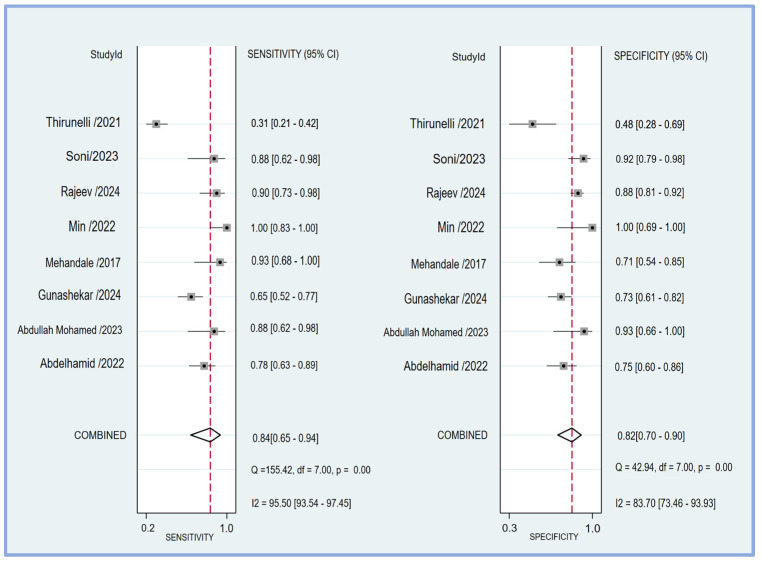
The forest plot showing the pooled sensitivity and specificity of the perfusion index (PI) in predicting post-induction hypotension [17,18,19,20,21,23,24,25].

**Figure 5:**
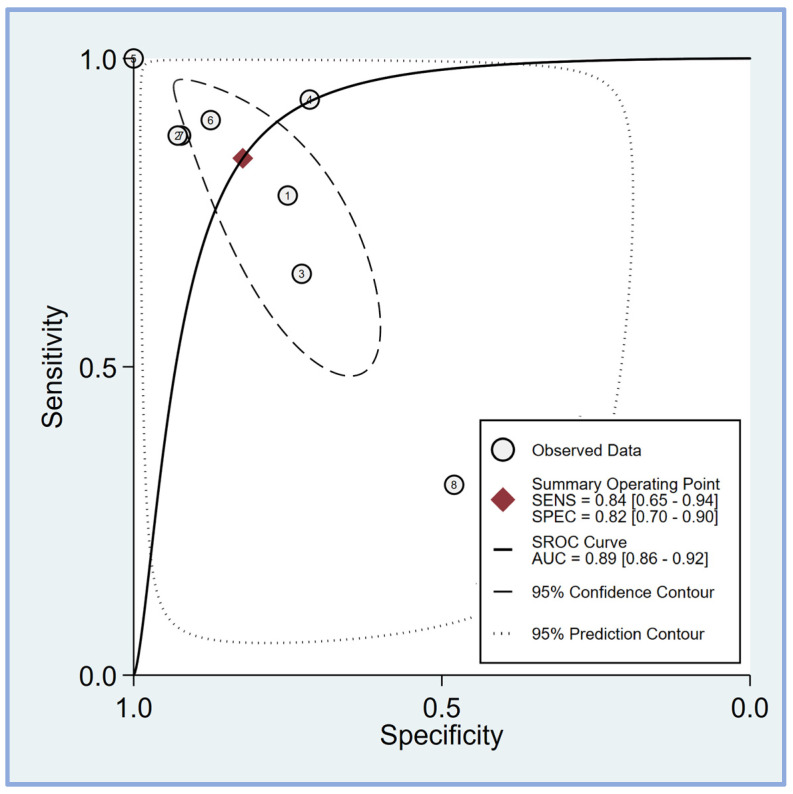
The summary receiver operating characteristic (sROC) curve analysis demonstrates the effectiveness of the perfusion index (PI) in predicting post-induction hypotension [17,18,19,20,21,23,24,25]. The weighted sROC curve is shown as a solid line, with individual study estimates of sensitivity and (1-specificity) represented by open circles. Combined results across studies are indicated by diamonds, representing pooled point estimates of outcomes. AUC stands for the area under the curve, while SENS and SPEC refer to sensitivity and specificity, respectively.

The pooled sensitivity and specificity of the perfusion index for predicting PIH in patients undergoing general anesthesia were analyzed in eight studies [17,18,19,20,21,23,24,25]. The sensitivity across the studies showed considerable variability, ranging from 0.31 (95% CI: 0.21–0.42) to 1.00 (95% CI: 0.83–1.00). The combined sensitivity was calculated to be 0.84 (95% CI: 0.65–0.94) (Figure 4), indicating a high ability of the perfusion index to correctly identify patients who would develop hypotension post-induction. Similarly, the specificity varied among the studies, with values ranging from 0.48 (95% CI: 0.28–0.69) to 1.00 (95% CI: 0.69–1.00). The combined specificity was determined to be 0.82 (95% CI: 0.70–0.90) (Figure 4), demonstrating a strong capability of the perfusion index to correctly identify patients who would not develop hypotension post-induction. The heterogeneity among the studies was significant for both sensitivity (I^2^ = 95.50 [95% CI: 93.54–97.45]) and specificity (I^2^ = 83.70 [95% CI: 73.46–93.93]), indicating substantial variability in the results across different studies. The predictive efficacy of the perfusion index for PIH was further evaluated using the pooled AUC from the sROC curve. The sROC analysis revealed an AUC of 0.89 (95% CI: 0.86–0.92) (Figure 5), demonstrating a high overall accuracy of the perfusion index in predicting PIH.

#### 3.2.3. Fagan’s Nomogram Analysis

**Figure 6:**
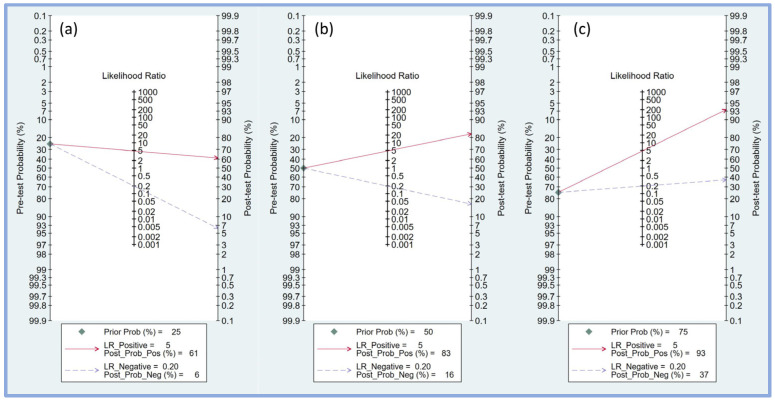
Fagan’s nomogram plot displaying the relationship between pre-test probability, likelihood ratio, and post-test probability at varying prior probabilities of 25% (**a**), 50% (**b**), and 75% (**c**), respectively [17,18,19,20,21,23,24,25]. LR, likelihood ratio; Prob, probability; Pos, positive; Neg, negative.

Fagan’s nomogram was employed to assess the clinical utility of the perfusion index in predicting PIH by combining pre-test probabilities with likelihood ratios to estimate post-test probabilities. For a pre-test probability of 25% (Figure 6a), a positive likelihood ratio of 5 resulted in a post-test probability of 61%, indicating that when the perfusion index predicted hypotension, the likelihood of PIH increased to 61%. Conversely, a negative likelihood ratio of 0.20 decreased the post-test probability to 6%, showing a substantial reduction in the likelihood of PIH when the perfusion index did not indicate hypotension.

With a pre-test probability of 50% (Figure 6b), the positive likelihood ratio of 5 increased the post-test probability to 83%, significantly increasing the probability of PIH when the perfusion index was positive. A negative likelihood ratio of 0.20 lowered the post-test probability to 16%, indicating a notable reduction in the risk of PIH when the perfusion index was negative.

For a higher pre-test probability of 75% (Figure 6c), the positive likelihood ratio of 5 increased the post-test probability to 93%, indicating a very high likelihood of PIH when the perfusion index was positive. A negative likelihood ratio of 0.20 reduced the post-test probability to 37%, indicating that while the likelihood of PIH is lower, it remains substantial even when the perfusion index does not indicate hypotension.

#### 3.2.4. Deek’s Funnel Plot Asymmetry Test

**Figure 7:**
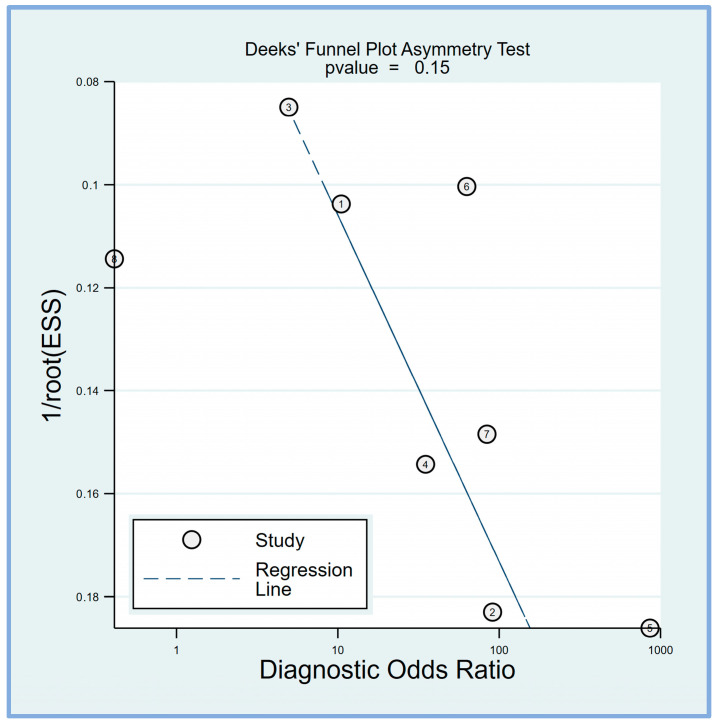
Deek’s funnel plot asymmetry test was conducted to assess the presence of publication bias among the included studies, revealing a low risk of bias (*p* = 0.15) [17,18,19,20,21,23,24,25].

Deek’s funnel plot asymmetry test was performed to evaluate potential publication bias in the studies included in this meta-analysis. Deek’s funnel plot asymmetry test suggested that the results are unlikely to be significantly influenced by publication bias (*p* = 0.15) (Figure 7).

## 4. Discussion

The systematic review and meta-analysis evaluated the predictive efficacy of the perfusion index for PIH in patients undergoing general anesthesia. The pooled results demonstrated a high incidence of PIH and a strong predictive performance for the perfusion index, with high sensitivity (i.e., 0.84), specificity (i.e., 0.82), and AUC values (i.e., 0.89). Fagan’s nomogram analysis showed that the perfusion index significantly altered the post-test probabilities of PIH across various pre-test probability scenarios. The Deek funnel plot asymmetry test revealed no significant publication bias.

The perfusion index is a noninvasive measure derived from photoplethysmography, a technique widely used in intraoperative monitoring. By quantifying the ratio of pulsatile to non-pulsatile blood flow, this index can serve as a valuable indicator of perfusion status [26,27]. Notably, the perfusion index may detect changes in central blood volume earlier than traditional measures, such as mean arterial pressure [28], potentially offering a more sensitive tool for hemodynamic management. Research has shown that lower perfusion index values correlate with poorer outcomes in both surgical patients and critically ill individuals [29,30]. In the operating room setting, increasing evidence suggests that the use of the perfusion index may help predict the occurrence of acute kidney injury postoperatively [31,32]. Given its ability to provide rapid, noninvasive insights into a patient’s perfusion status, the perfusion index may emerge as a promising parameter for optimizing perioperative care and potentially reducing postoperative complications. Nevertheless, the efficacy of the perfusion index in predicting PIH remains unclear and no systematic approach has been conducted to evaluate its effectiveness.

Our meta-analysis provides evidence supporting the efficacy of the perfusion index as a noninvasive predictor of PIH in patients receiving propofol for induction. These findings suggest that the perfusion index could serve as a valuable screening tool to identify patients at high risk of PIH, enabling anesthesiologists to implement targeted preventive strategies and optimize perioperative management. The high sensitivity of the perfusion index indicates its ability to correctly identify a significant proportion of patients who will develop PIH, while its high specificity suggests that it can accurately rule out patients who are unlikely to experience PIH. An AUC of 0.89 further supports the overall diagnostic accuracy of the perfusion index in predicting PIH. 

The clinical utility of the perfusion index was further demonstrated by Fagan’s nomogram analysis, which showed that a positive perfusion index result substantially increased the post-test probability of PIH, whereas a negative result decreased the likelihood. This suggests that incorporating the perfusion index into the preoperative risk assessment could help guide clinical decision-making and resource allocation. For example, patients with a high perfusion index could be targeted for more aggressive hemodynamic monitoring, fluid optimization, and vasopressor use, whereas those with a low perfusion index may require less intensive interventions. The perfusion index offers several advantages as a predictive tool for PIH. It is a noninvasive, readily available, and easily interpretable parameter that can be obtained from standard pulse oximetry monitoring. Unlike other predictive methods, such as invasive hemodynamic monitoring or advanced echocardiographic techniques [33,34], the perfusion index does not require additional equipment or expertise, making it potentially more accessible and cost-effective in clinical practice. However, the perfusion index should not be considered a standalone predictor of PIH but rather a complementary tool that can be integrated with other clinical parameters and risk factors. Factors such as age, comorbidities, medications, and fluid status may influence the occurrence of PIH [15,35] and should be considered in conjunction with the perfusion index when assessing PIH risk.

The present meta-analysis revealed a high incidence of PIH in patients undergoing general anesthesia, with a pooled event rate of 44.8%. This finding highlights the significant risk of PIH in the perioperative setting, even among relatively healthy patients with an ASA physical status of I-II. The high incidence of PIH observed in this meta-analysis is consistent with previous reports, which have documented rates ranging from 18.1% to 70% [10,11]. The wide variation in PIH incidence across individual studies may be attributed to differences in patient populations, anesthetic techniques, and the definitions of hypotension used. Notably, all included studies utilized propofol for anesthesia induction, with doses ranging from 1 to 2 mg/kg or titrated to effect. Propofol, a commonly used intravenous anesthetic agent, is known to cause significant hypotension due to its vasodilatory and myocardial depressant effects [36,37]. The high incidence of PIH associated with propofol use underscores the need for careful hemodynamic monitoring and management during anesthesia induction, even in relatively low-risk patients. Strategies such as slow titration of propofol, preemptive fluid optimization, and judicious use of vasopressors may help mitigate the risk of PIH in this population [38,39,40,41]. However, further research is needed to establish an optimal approach to prevent and manage PIH in patients undergoing general anesthesia with propofol.

The meta-analysis by Liu et al. demonstrated that several preoperative ultrasound measurements, including the inferior vena cava collapsibility index (IVC-CI), maximum and minimum inferior vena cava diameters (DIVCmax and DIVCmin), and carotid artery-corrected flow time (FTc) showed good predictive accuracy for PIH [42]. Among these parameters, the carotid artery FTc had the highest AUC of 0.91, with a pooled sensitivity of 0.81 and specificity of 0.87, suggesting that it may be the most accurate ultrasound measurement for identifying patients at risk of PIH [42]. The authors also found that factors such as age, cutoff values, and anesthetic agents could influence the predictive accuracy of these ultrasound parameters [42]. Our meta-analysis indicated that the perfusion index exhibited a strong predictive performance for PIH, with pooled sensitivity, specificity, and AUC values of 0.84, 0.82, and 0.89, respectively. The evidence from these meta-analyses supports the efficacy of both preoperative ultrasound measurements and the perfusion index in predicting PIH in patients undergoing general anesthesia. The advantage of the perfusion index is that it is readily available in most operating rooms and, unlike ultrasound measurements, does not require additional equipment or expertise.

This meta-analysis has several limitations that should be considered when interpreting the results. First, the included studies exhibited significant heterogeneity in both sensitivity and specificity estimates, which may be attributed to differences in patient populations, anesthetic techniques, and the definitions of PIH. Second, the optimal cutoff values for the perfusion index varied widely across studies, making it challenging to establish a universally applicable threshold for predicting PIH. Third, the majority of the included studies focused on patients with ASA physical status I-II, which may limit the generalizability of the findings to higher-risk populations. Fourth, the included studies did not consistently report potential confounding factors, such as comorbidities, medications, and fluid management, which could influence the occurrence of PIH and the predictive performance of the perfusion index. Despite these limitations, this meta-analysis provides valuable insights into the predictive efficacy of the perfusion index for PIH and highlights the need for further research to address the identified gaps in knowledge.

## 5. Conclusions

The high sensitivity, specificity, and AUC of the perfusion index suggest that it could serve as a valuable screening tool to identify patients at high risk of PIH, enabling anesthesiologists to implement targeted preventive strategies and improve patient safety. However, the considerable heterogeneity among the included studies underscores the necessity for further research to identify potential sources of variability and refine the optimal cut-off values for the perfusion index. More studies are required to assess the impact of incorporating the perfusion index into clinical practice on patient outcomes and healthcare costs.

## Figures and Tables

**Table 1:** Search strategy for MEDLINE.

1	(“General anesthesia” or “Inhalation agents” or “Propofol” or “Total intravenous anesthesia” or “Tracheal intubation” or “Sevoflurane” or “Isoflurane” or “desflurane”).mp.
2	exp “Anesthetics, General”/
3	(“Perfusion index” or “PI” or “Peripheral perfusion index”).mp.
4	exp “Perfusion Index”/
5	(“Hypotension” or “Low Blood Pressure” or “Reduced blood pressure”).mp.
6	exp “Hypotension”/
7	(1 or 2) and (3 or 4) and (5 or 6)

**Table 2:** Characteristics of studies (*n* = 8).

Studies	Age (Years)	Sex (M/F)	N	ASA	PI Cut-Off Value	AUC	Country
Abdelhamid 2022 [20]	31	49/44	93	I-II	<3.03	0.776	Egypt
Abdullah Mohamed 2023 [17]	71	19/11	30	I-II	≤1.3	0.97	Egypt
Gunashekar 2024 [23]	40	83/57	140	I-II	<3.5	0.647	India
Mehandale 2017 [24]	31	29/21	50	I-II	<1.05	0.816	India
Min 2022 [25]	66–68	17/13	30	II	≤0.96	1	Korea
Rajeev 2024 [18]	39	60/114	174	I-II	<2.45	0.8793	India
Soni 2023 [19]	40	27/28	55	I-II	<1.03	0.913	India
Thirunelli 2021 [21]	42	53/53	106	I-II	<1.05	0.511	India

AUC: area under curve; PI: perfusion index; ASA: American Society of Anesthesiologists physical status.

**Table 3:** Induction agents and details of post-induction hypotension.

Studies	Induction Agent (Propofol)	PIH (%)	Definition of Hypotension	Follow-Up
Abdelhamid 2022 [20]	1.5–2 mg/kg	48.40%	dMAP > 25% baseline	na #
Abdullah Mohamed 2023 [17]	1–2 mg/kg	53.30%	dSBP of >30% of baseline	3 min
Gunashekar 2024 [23]	2 mg/kg	45%	dSBP of >30% of baseline	5 min
Mehandale 2017 [24]	10 mg per every 5 s †	30%	dSBP of >30% of baseline or MAP < 60 mmHg	5 min
Min 2022 [25]	3.0 μg/mL ⁋	66.70%	SBP < 90 mmHg	15 min
Rajeev 2024 [18]	10 mg per every 5 s †	17.20%	MAP < 60 mmHg	5 min
Soni 2023 [19]	10 mg per every 5 s †	29%	dSBP > 30% of baseline or MAP < 60 mmHg	5 min
Thirunelli 2021 [21]	2 mg/kg	76.40%	dMAP > 20% of baseline or MAP < 60 mmHg	5 min

⁋ target effect-site concentration; † titrated to loss of consciousness; # period from induction of anaesthesia until surgical stimulation; dMAP: drop in mean arterial blood pressure; dSBP: drop in systolic blood pressure; PIH: post-induction hypotension.

## Data Availability

The original contributions presented in this study are included in this article. Further inquiries can be directed to the corresponding authors.
